# The Treatment of Incipient Insanity

**Published:** 1906-02

**Authors:** A. D. Young

**Affiliations:** Oklahoma City, Okla; Professor of Neurology and Psychiatry, Medical School, Epworth University; Consulting Neurologist, St. Anthony’s Hospital; Formerly with St. Louis Insane Asylum and Illinois Central Insane Hospital


For Texas Medical Journal.

### The Treatment of Incipient Insanity.

A. D. YOUNG, M. D., OKLAHOMA CITY, OKLA.

Professor of Neurology and Psychiatry, Medical School, Epworth University; Consulting Neurologist, St. Anthony's Hospital; Formerly with St. Louis Insane Asylum and Illinois Central Insane Hospital.

The prevalent idea that every case of insanity is practically incurable, to my mind, has been the cause of many persons becoming hopelessly insane. Naturally the general practician is the first to see the acute cases, and he is usually consulted while the attack is yet in its incipiency; but being a very busy man and not having the time to keep in touch with the more advanced treatment of a trouble so seldom encountered, the patient is denied the benefit of early and rational treatment. This is unfortunate, as much good can be accomplished if these cases of beginning mental aberration are intelligently managed in the very commencement.

Should he send these cases to the alienist? Assuredly no; if he has the time, patience and experience to devote to them the attention they deserve. If not, then, most assuredly, yes. It is for the former this article is written.

The first requisite in the management of these cases is a good nurse, preferably one experienced in this line of work. Without such a one we can not hope to have the necessary control of the patient, nor to have a regular course of treatment carried into effect. A good cook is essential in furnishing a proper diet. Otherwise our efforts to increase bodily nutrition will be in vain.

The condition most frequently encountered in what may be called the curable cases of insanity, is one of mal-nutrition. The patient is pale and anemic. The diet must be abundant and of the most digestible kind. It is astonishing how much nourishment a patient can sometimes take without disturbance of the digestive organs. Milk and egg albumen are probably the best of the foods for forced feeding. When there is no organic lesion of the brain, whisky or wine may be added with benefit. Vegetables and fruits, especially those with small amounts of starch, should be given freely, while the various meat broths are suitable and serviceable. Many melancholiacs and some maniacs refuse to eat, and must be fed forcibly by means of the stomach tube with funnel attached, or by means of a tube passed through the nose and thus into the œsophagus. It is needless to say care must be exercised to see that the tube is properly introduced. I have never had much success with rectal feeding,—probably because of faulty technique.

In addition to forced feeding, the ordinary bitter tonics should be administered to stimulate the digestive organs to their greatest activity, and some form of organic iron should be given in conjunction with them. The latter answers the purpose much better than the usual inorganic combinations. As a supplement to these various measures, the numerous forms of baths may be used to meet special indications.

Strict confinement to bed, as a rule, is not beneficial and may do harm. However, it may be necessary for patients who are very weak. As for violent maniacs, it is better to send them to the country in charge of competent attendants where they can enjoy the open air and be diverted and amused in a quiet way. These measures alone will shorten many cases of acute mania, and the patients are never so violent as when confined.

Sedatives should never be used unless other measures fail, as they are seldom beneficial to the future course of the disease, and even at times retard recovery. When it is necsssary to use them, nothing is better than trional, although chloral is nearly as good. The former should be given about three hours before bedtime, as it is slow in its action. In cases characterized by great restlessness and motor disturbance, hyoscine hydrobromate, given hypodermatically in small doses, is a good remedy.

The question always arises: "Should the patient be treated at home or be placed in a proper institution?" I would answer, if the family is one with means so that proper attendants, suitable food, and change of surroundings can be readily obtained; if there is no homicidal nor suicidal tendencies, they will do better at home. Otherwise they should be placed where these measures can be successfully carried into effect.

---

Of course, Lambert won out. The Lewis & Clark Exposition at Portland last July gave them the gold medal for their products. See Lambert's ad on first ad page, front.

